# Consumer Attitudes and Preferences for Healthy Boxed Meal Attributes in Taiwan: Evidence from a Choice Experiment

**DOI:** 10.3390/nu15041032

**Published:** 2023-02-19

**Authors:** Min-Yen Chang, Jui-Chi Lin, Han-Shen Chen

**Affiliations:** 1 Department of Accounting, Jiaxing University, Jiaxing 314001, China; 2 Department of Health Industry Technology Management, Chung Shan Medical University, Taichung City 40201, Taiwan; 3 Department of Medical Management, Chung Shan Medical University Hospital, Taichung City 40201, Taiwan

**Keywords:** traceable agricultural products, carbon footprint, consumer behavior, willingness to pay, sustainable development goals

## Abstract

Consumers have realized the importance of a healthy diet, hoping to reduce the occurrence of obesity and chronic diseases. Therefore, healthy boxed meals with low calories and high protein are gaining attention. This study divided the attributes of healthy boxed meals into five categories, namely, taste, nutrition facts, traceability certification, carbon footprint label, and price, and investigated the relationship between consumer preferences and willingness to pay (WTP) using a choice experiment. A purposive sampling procedure was used to collect 495 valid questionnaires. The results indicate the following: (1) when purchasing healthy boxed meals, the respondents were most concerned with traceability certification and nutrition facts; (2) the respondents were willing to pay a premium for meals with traceability certification (NTD 4.6) and nutrition facts (NTD 4.4); (3) respondents > 40 years with an average monthly salary of > NTD 30,000 who worked out regularly and were in the process of muscle building, fat loss, or weight control had higher WTP for meals with nutrition labels; and (4) female respondents who were 30–39 years old with a college or university education or above had higher WTP for meals with traceable ingredients. The results may help healthy boxed meal companies understand and pay attention to consumer needs, which will, in turn, provide a reference for future product development and marketing strategies.

## 1. Introduction

The employment rate of women has increased in Taiwan with economic and social development, while the frequency of cooking at home has decreased and dietary habits have changed, leading to a yearly increase in the number of people who eat out. According to the 2019 Dining Trends in Taiwan published by Ipsos [1], as many as 70% of Taiwanese people eat outside the home or use takeout more than seven times a week, and more than 20% eat outside the home three meals a day. Ninety percent of Taiwanese food eaters choose to eat out with boxed meals, which has led to a booming bento industry in Taiwan [2]. Boxed meal means that the staple food is rice, noodles, or other staple food as the main raw material, accompanied by agricultural, livestock, aquatic products and other cooking dishes, which are combined (or mixed and stir-fried), properly packaged, and sold in a short time, for consumers to immediately eat food [3].

The convenience of food deliveries makes it possible for consumers to obtain delicious meals quickly. According to the Health Promotion Administration of the Ministry of Health and Welfare (MOHW) (2019) [4], generally, adult women require about 1500–1800 kcal a day, while men require about 2000–2300 kcal. People consume too many calories and fat in their daily diets, leading to a yearly rise in chronic diseases, such as obesity, diabetes, and cardiovascular disease. According to a survey on eating outside the home in Taiwan, about 80% of the respondents expect that the meals they buy should also be healthy, and up to 87% of them expect that takeout meals should be labeled with complete nutritional information [5]. In response to this market trend, some boxed meal companies have begun to focus on healthy boxed meals with simple cooking methods, low oil, and high fiber and disclose complete nutritional information on the boxes for people’s reference and choice.

Muscle Beach was the first company to enter the healthy boxed meal business in 2014. Subsequently, Miss Energy, The Protein Box, Health It, and other chain healthy boxed meals companies were established in response to market demand. In addition to taste, consumers were beginning to attach importance to whether the caloric and nutritional content of boxed meals met their needs.

As consumers’ requirements for eating out have increased significantly, caterers are paying more attention to the characteristics of meals that affect consumers’ willingness to purchase them, such as brand, service attitude, and nutrition of meals. Previous studies have been conducted on consumer food preferences, such as brand and price [6], nutrition facts [7], taste preference [8], and food safety [9]. In the past, studies related to boxed meals mainly focused on taste, nutrition facts, and price. There are still important research gaps that need further exploration. In the era of open and transparent food safety information, it is advantageous to advertise traceable ingredients; in addition, with increasing consumer awareness and global emphasis on energy conservation and carbon reduction, carbon footprint label is bound to be one of the trends for the future. Therefore, in addition to the above three attributes, this study adds the attributes of traceability certification and carbon footprint label to build a more complete integrated model, in order to find possible explanations and fill this important research gap. The results of this study can be used to analyze and explain the factors that influence consumers’ purchase of healthy boxed meals. More importantly, business managers in the catering industry can understand the key influencing factors for consumers’ purchases to formulate effective strategies, increasing the practical benefits and application value of marketing, which is an important contribution of this study.

## 2. Literature Review

The following explains the implications of various product attributes:

### 2.1. Taste

Li and Zhu [10] suggested that freshness, nutritional value, and taste serve as the intrinsic indicators of food consumption perceived quality. Petrescu et al. [11] indicated that consumers most frequently use freshness, taste, and appearance to evaluate food quality. Livingstone et al. [12] explored the dietary preferences of young adults in the USA by studying attributes such as nutritional content, cost, taste, familiarity, and preparation time. The results revealed that nutritional content was the most important influence on meal choices, followed by cost, taste, familiarity, and preparation time. In a Dutch study on food choice motivation, taste was found to be the dominant motivation across time, place, and social context [13].

### 2.2. Nutrition Facts

Nutrition facts provide consumers with nutritional information that helps them make informed and healthy food choices [14,15,16,17,18]. Wojcicki and Heyman [19] indicated that US adolescents made use of the information on the nutrition facts label, with the highest percentage using the total fat on the nutrition facts label. Crockett et al. [20] indicated that consumers can use nutrition facts to understand product information, choose foods, and organize their diet according to their needs. Shangguan et al. [21] indicated that consumers pay attention to the information on food labels before purchase, and their knowledge about nutrition facts affects their purchase intention.

According to Meijer et al. [22], if food products are clearly labeled with their nutritional content, consumers’ understanding of nutrition facts can be enhanced and their choice of unhealthy foods can be reduced, decreasing the incidence of obesity; in addition, food business operators can reduce the levels of added sugars, salt, and saturated fats in foods, and discontinue the use of partially hydrogenated oils and fats in response to market demand. The above studies indicate that nutrition facts of food products affect people’s choice of food and have positive effects on health promotion, disease prevention, and control.

### 2.3. Traceability Certification

As several food safety incidents have occurred around the world, several countries have established food traceability certification to provide information about the process from farm to fork, reducing uncertainty when purchasing food and enhancing consumers’ trust and willingness to pay (WTP) [23,24]. Hong et al. [25] mentioned that consumers’ primary considerations when shopping for boxed meals are hygiene, food safety, and freshness. Ortega et al. [26] applied the choice experiment method (CE) to assess Beijing consumers’ WTP for selected food quality attributes (food safety, animal welfare, green food, and organic certification), taking into account country of origin information. Results show that Beijing consumers place the highest value on food safety information and are willing to pay higher prices for Australian beef products than American or domestic (Chinese) beef. Wongprawmas and Canavari [27] noted that consumers in Thailand are willing to pay a premium for agricultural products with food safety labels.

According to Kumvenji et al. [28], the food traceability system enhances people’s confidence in food safety because of its clear records of traceability. Nguyen et al. [29] explored the preferences and willingness to pay (WTP) of consumers in the USA for high-end restaurant meals and found that consumers are willing to pay higher prices for meals that are sourced transparently. As it has become a trend in the food industry to make information transparent by disclosing the source of food ingredients, the use of traceability-certified ingredients not only enables consumers to trace the source of ingredients but also serves as a way for restaurants to manage food safety. Therefore, whether the WTP of consumers for healthy boxed meals will be affected when they are labeled and use traceable ingredients deserves further exploration.

### 2.4. Carbon Footprint Label

With the increasing awareness of environmental protection and the global trend toward energy conservation and carbon reduction, consumers are increasingly concerned about the daily environmental impact on their lives [30,31]. Smith et al. [32] specified that food production and consumption is one of the key factors in global climate change. In response to global climate change, the United Nations proposed the 2030 Sustainable Development Goals (SDGs) in 2015. Achieving net-zero emissions has become a common goal for all countries. In March 2022, “Taiwan’s Pathway to Net-Zero Emissions in 2050” was officially announced to implement the goal of net-zero transformation. In the same year, the Food and Agricultural Education Act was passed to support the development of sustainable agriculture, including local agriculture, food waste reduction, and eat local first.

Research in Egypt found that consumers who are accustomed to buying green products are willing to pay a premium for a carbon footprint label [33]. Grasso and Asioli [34] suggested that the use of a carbon footprint label on sustainable food can increase the WTP for consumers in the UK. However, Colantuoni et al. [35] indicated that on potatoes, the presence of a carbon footprint label reduced the German and Italian total WTP, while the ethical certification was considered very important. In addition, studies have found that gender, age, education, income, and region of origin affect consumer perceptions of foods with a carbon footprint label [36,37].

### 2.5. Choice Experiment

Consumer choices of products involve elements such as consumer perceptions, expectations, social and psychological factors, financial environment, and intrinsic or extrinsic product characteristics [38]; price is the determinant of extrinsic product characteristics affecting purchase intention [39]. When consumers have a positive concern for the environment, they are willing to pay a higher amount for goods that are less harmful to the environment and engage in environmentally friendly behaviors [40]. Rex and Baumann [41] pointed out that consumers are willing to pay higher prices for green products.

In exploring consumers’ WTP for green products, the choice experiment method (CE), with its ability to evaluate multiple attributes and levels, can combine different alternatives for important characteristics related to nonmarket goods or services, and enables respondents to state their preferences for hypothetical alternative scenarios. This method has been widely used in the fields of consumer behavior [42,43], sharing economy [44,45], green energy [46,47], tourism, and leisure [48,49]. In recent years, it has been gradually applied in the food industry [6,50,51,52].

Liu et al. [53] applied the choice experiment method (CE) to construct a utility model for coffee certification attributes. The results of their study indicate that respondents’ WTP for attributes ranked from highest to lowest includes traceability, organic, graded, environment friendly, and fair-trade certifications. Rusmevichientong et al. [52] applied the choice experiment method (CE) to explore the preferences of 11- to 13-year-old middle school students in California, USA, for snack choices. The attributes studied included price, nutrition, socialization, taste, and convenience. The findings indicate that price is the most important factor for respondents, while convenience is the least important. Chang et al. [50] applied the choice experiment method (CE) to explore Taiwanese consumers’ choices and WTP for yogurt. The attributes studied included the number of probiotic species, fermented milk sources, edible colloids, and healthy food labels. The results indicate that consumers placed the most importance on healthy food label, followed by the number of probiotic species.

## 3. Materials and Methods

### 3.1. Survey Design

To avoid questionnaire errors and inaccurate analysis results, it’s important to choose attributes for healthy boxed meals that align with consumers’ purchase motivation. Some healthy boxed meal manufacturers in Taiwan have already incorporated attributes such as nutrition facts, traceability certification, and carbon footprint label. For this reason, after referring to and integrating previous literature, this study included consumers’ consideration of purchasing healthy-boxed-meals-related motivations into the main attributes of the discussion, namely, taste (consumers’ self-centered taste) and nutrition facts (providing nutrition facts for meals). In addition, add traceability certification (use- and non-use-related verification materials) and a carbon footprint label (restaurants with verification and no verification) to measure the willingness to pay the price. This study set the reference point of the carbon footprint label to 32 g CO_2_ for healthy boxed meals. Where to set these cut-off levels in practice is obviously an important question, which should be settled based on technical expertise. This study only provides evidence regarding the principle: whether adding information about the carbon footprint of healthy boxed meals, in addition to the amount of carbon emission through the healthy boxed meal, makes the label more intuitively understandable for common consumers and therefore more effective.

The price levels were determined based on the pricing (NTD 99) of healthy boxed meal companies, such as The Protein Box and Health It chain healthy boxes, reflecting a realistic price range in the studied market at the time of the study. The following items are provided in the pretest questionnaire: (1) If the healthy boxed meals provide complete nutrition facts, how much extra are you willing to pay? (2) If the healthy boxed meals use traceable ingredients, how much are you willing to pay extra? (3) If the healthy boxed meals provide carbon footprint certification, how much extra are you willing to pay? The above statistical results show how much extra they are willing to pay for the total amount (NTD 0 extra, NTD 1–5 extra, NTD 6–10 extra) with the highest proportion, so the three levels are provided for the respondents to fill in the formal questionnaire, as shown in Table 1.

**Figure 1:**
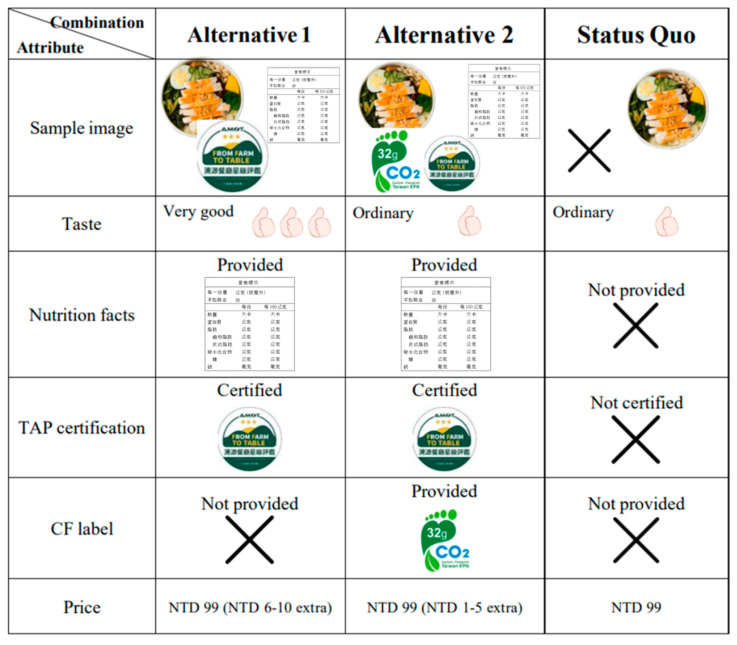
Example questionnaire choice set.

Based on the above attributes and attribute levels, we obtained 72 (3 × 2 × 2 × 2 × 3) combinations. It is impossible for respondents to make a decision when facing too many options because of their bounded rationality. To address this problem, an available way is to extract representative ones by using an orthogonal design combined with the IBM SPSS Statistics 25.0 software. In this paper, 16 alternative options were produced by using the orthogonal experimental design. The 16 alternative options were mixed at random and then paired into 5 sets. Each choice set was made up of two alternative options and one status option. Respondents would belong to one of the three groups at random, and each of them was provided with 3 choice sets. Respondents were required to make a decision among every three options, and then they needed to answer some other questions in regard to their personal information. One example of the choice sets can be seen in Figure 1.

The formal questionnaire was divided into four parts. The first part was used to understand the respondents’ experience in purchasing healthy boxed meals, the number of times they purchased each week, and their exercise habits. The second part was used to find how much the respondents value each attribute of healthy boxed meals (all the questions were based on a 5-point Likert scale, according to the respondents’ perceptions or actual situations, with a score of 1 representing “strongly disagree” and 5 “strongly agree”). The third part investigated the respondents’ preferences for the attributes of healthy boxed meals, with each choice set containing three scenarios, one for the current situation and two for the filtered design (see Figure 1). The fourth part investigated the respondents’ socioeconomic background, including their gender, age, education level, average monthly income, number of times eating out per week, daily spending on eating out, and body mass index (BMI). According to the Health Promotion Administration, MOHW (2021) [55], the weight of adults over 18 years of age in Taiwan is classified into underweight (BMI < 18.5), healthy (18.5 ≤ BMI < 24), overweight (24 ≤ BMI < 27), and obese (BMI ≥ 27). For adult waist circumference, male waist circumferences are classified into healthy (<90 cm) and obese (≥90 cm), and female waist circumferences are classified into healthy (<80 cm) and obese (≥80 cm). Waist circumference is often used as a simple measure for the risk of developing metabolic syndrome and cardiovascular disease.

### 3.2. Choice Analysis: Conceptual Framework and Statistical Model

The choice experiment method (CE) is a hypothetical method of stating preferences derived from Lancaster’s consumer theory [56]. As the choice experiment method (CE) considers multiple attributes in decision making, it can be used to identify interactions between attributes and to compare the relative importance of attributes [57]; furthermore, it can present trade-offs between attributes that respondents must consider when making decisions [58]. First, we applied the choice experiment method (CE) to construct a utility model for healthy boxed meal attributes; second, we applied the random parameter logit (RPL) models to estimate the utility function of healthy boxed meals, and explored the differences in the WTP for each attribute from the perspective of the respondents’ perceptions and behaviors.

As respondents with the same socioeconomic characteristics may have different preferences for the attributes of healthy boxed meals, the random parameter logit (RPL) model needed to be estimated. It is generally believed that consumers prefer lower prices; therefore, price preferences remain isomorphic across consumers [59]. The formula for the random parameter logit (RPL) model is shown in Equation (1):

(1) $$U_{ij}=V_{ij}+\varepsilon_{ij}=V\left(H_{j}\right)+\varepsilon_{ij}$$

where $U_{ij}$: the utility of the *i*-th respondent for the attribute combination 𝐻𝑗 of the *j*-th product; 𝑉_𝑖𝑗_: the measurable utility of the *j*-th product for the *i*-th respondent, which is observable; 𝐻_𝑗_: the vector of attribute in the choice set; and 𝜀_𝑖𝑗_: the random error, which is unobservable.

Assuming that the measurable utility for respondents is a linear additive model, Equation (2) is given as follows:

(2) $$U_{ij}=V_{ij}+\varepsilon_{ij}=\sum_{k=1}^{K}\;\alpha_{k}X_{jk}+\beta P_{j}+\varepsilon_{ij}$$

where 𝑘 = 1, 2, …, 𝐾; 𝑋_𝑗𝑘_: the attribute k of the *j*-th product in the choice set; 𝑃_𝑗_: the price attribute of the *j*-th product; 𝛼_𝑘_, 𝛽: the coefficient of attribute/variable; and 𝜀_𝑖𝑗_: the random error, which is unobservable.

To compare the differences in product preferences among different groups of consumers, the product attributes in the indirect utility function should be cross-tabulated with the socioeconomic characteristics of the respondents. As the socioeconomic characteristics are fixed and do not change due to product selection, they cannot be added to the indirect utility function [60]. Therefore, Equation (3) is modified as follows:

(3) $$U_{ij}=\overset{K}{\underset{k=1}{\sum }}\;\alpha_{k}X_{jk}+\beta P_{j}+\overset{K}{\underset{k=1}{\sum }}\;\overset{M}{\underset{m=1}{\sum }}\;\gamma_{km}X_{jk}D_{im}+\overset{M}{\underset{m=1}{\sum }}\;\gamma_{pm}P_{j}D_{im}+\varepsilon_{ij},$$

where 𝑈_𝑖𝑗_: the utility of the *i*-th respondent for the attribute combination 𝐻𝑗 of the *j*-th product; 𝑃_𝑗_: the price attribute of the *j*-th product; 𝛼_𝑘_, 𝛽: the coefficient of attribute; 𝐷_𝑖𝑚_: the *i*-th respondent’s *m*-th socioeconomic characteristic; 𝛾_𝑘𝑚_ and 𝛾_𝑝𝑚_: the coefficients of the interaction term between the attribute with socioeconomic characteristics and price; 𝜀_𝑖𝑗_: the random error, which is unobservable; and 𝑖 = 1, 2, …, 𝐼; 𝑗 = 1, 2, …, 𝐽; 𝑘 = 1, 2, …, 𝐾; 𝑚 = 1, 2, …, 𝑀.

The above model can calculate the WTP at different attribute levels by dividing the marginal utility of each attribute by the marginal utility of price and taking the negative value of the marginal rate of substitution [61,62]. WTP is calculated as shown in Equation (4):

(4) WTP=−∂V∂Xk∂V∂P=−βkβP

where *V* is the measurable utility, *k* is the estimated coefficient value of the nonprice attribute, and *p* is the estimated coefficient value of the price attribute.

## 4. Results

### 4.1. Sample Size and Composition

In this study, we used the convenience sampling method for the survey questionnaire face-to-face in The Protein Box, Health It, and other chain healthy boxed meal companies in Taichung City, Taiwan. In consideration of research ethics, this study clearly informed the subjects on the front page of the questionnaire about the survey purpose of this study and participation in an anonymous form so that all subjects can provide their answers without worrying about privacy.

First, the study conducted a pretest questionnaire, with the aim of understanding consumers’ overall consumption preferences and WTP for healthy boxed meals. The questionnaires were issued from January 2022 to February 2022 to consumers who had purchased healthy boxed meals. A total of 92 effective samples were recovered in the pretest of the questionnaire. The formal questionnaire was distributed and remained available from March 2022 to April 2022; the main target population was “consumers who had purchased healthy boxed meals in the past 3 months.” A total of 495 valid questionnaires were collected, which are shown in Table 2.

From a demographic perspective, the majority of the respondents were female (54.9%). The age group was mainly concentrated among 20- to –29-year-olds (39.6%), followed by 30- to 39-year-olds (35.8%) and 40- to 49-year-olds (12.3%), indicating that consumers from younger age groups have more experience in purchasing healthy boxed meals than those from other groups. In terms of education level, the highest percentage of consumers were college or university educated (70.9%). The average personal monthly income mainly ranged from NTD 30,001 to 50,000 (45.5%). The number of times eating out per week was mainly 9 times or more (33.5%), followed by 3–5 times (29.7%). The average daily spending on eating out was mostly less than NTD 200 (45.1%), followed by NTD 201–300 (38.2%). This result is consistent with that of the survey report by the Insight Xplorer Marketing Research Company (2018), which stated that individuals spend about NTD 246 per day on eating out. Most respondents (41%) purchased healthy meals less than once a week, followed by 1–2 times (36.2%).

In terms of weight control and exercise habits, a higher percentage of respondents worked out regularly (56.6%); most of them were engaged in muscle building and fat loss or weight control (64.4%). The majority of respondents (54.1%) had a BMI in the 18.5 ≤ BMI < 24 range, followed by 24 ≤ BMI < 27 (24.6%), which is similar to the average BMI of 24.2 for adults over 19 years old in the Nutrition and Health Survey in Taiwan 2013–2016 reported by the Health Promotion Administration (2019).

In addition, the most important attributes of healthy boxed meals were taste (4.42), followed by price (4.29), nutrition facts (4.1), and traceability certification (4.03), and the least important was carbon footprint label (3.37), indicating that the respondents tend to choose foods that are tasty and reasonably priced.

### 4.2. Consumers’ Preferred Combinations for Healthy Boxed Meals

According to the analysis results, the respondents’ preferred combination of attributes was “very good taste, with nutrition label, with traceability certification, without carbon footprint label, and a price of NTD 99 (NTD 6–10 extra)” (29.8%). The next most popular attribute combination was “very good taste, without nutrition label, with traceability certification, without carbon footprint label, and a price of NTD 99 (NTD 1–5 extra)” (20.14%). The respondents’ least preferred combinations were “good taste, without nutrition label, without traceability certification, with carbon footprint label, and a price of NTD 99 (NTD 1–5 extra)” and “good taste, with nutrition label, with traceability certification, with carbon footprint label, and a price of NTD 99 (NTD 6–10 extra),” accounting for 10.9% and 13.9% of the respondents, respectively.

Based on the results of the study, consumers value healthy boxed meals with traceability certification, which is consistent with Nguyen et al.’s [29] findings that consumers are willing to pay more for meals that are transparent about the source of ingredients; consumers also value healthy boxed meals with nutrition labels, which is consistent with Kang et al.’s [63] findings that consumers are willing to pay more for nutritious foods and Macdiarmid et al.’s [64] findings that consumers are willing to pay significantly more for products with nutrition labels than those with a carbon footprint label.

### 4.3. Results of the RPL Models

The coefficient values of each attribute in the random parameter logit (RPL) models were calculated using NLOGIT 4.0. The empirical estimation results are shown in Table 3. In particular, the random parameter logit (RPL) model estimated respondents’ differential preference for the attributes of healthy boxed meals. The coefficient value of status quo (ASC) was negative and significant at the 5% level, indicating that consumers were not satisfied with the attributes of healthy boxed meals currently available on the market.

In the random parameter logit (RPL) model, the coefficient value of carbon footprint label (CF) was significantly negative, indicating that the respondents placed less importance on having carbon footprint labels on healthy boxed meals, while very good taste (VGD) and traceability certification were significantly positive, indicating that respondents preferred these attributes.

Then, by substituting the coefficient values derived from Equation (1) of the utility function into Equation (4) of the theoretical model, the respondents’ WTP for each attribute could be calculated. The WTP for each attribute in the random parameter logit (RPL) model was NTD 3 for good taste (GD), NTD 4.3 for very good taste (VGD), NTD 4.4 for nutrition facts (NF), NTD 4.6 for traceability certification (TAP), and NTD 3.3 for carbon footprint label (CF).

### 4.4. Exploring the Differences in Willingness to Pay Based on Respondents’ Socioeconomic Background and Attributes of Healthy Boxed Meals

According to the analysis results of the random parameter logit (RPL) model, there were random parameters in nutrition facts (NF) and traceability certification (TAP). Thus, this study compared WTP based on these attributes, and the respondents’ socioeconomic backgrounds are shown in Table 4. Compared with other age groups, the respondents aged 20–29 and 30–39 years old were less likely to prefer the “status quo.” The WTP for nutrition facts demonstrated significant differences in age, average personal monthly salary, muscle building, fat loss or weight control, and regular exercise, with higher WTP among respondents over 40 years of age, indicating that younger respondents were less willing to pay extra for healthy boxed meals that provided nutrition facts. This is consistent with Ollberding et al.’s [65] findings, which indicate that people pay more attention to nutrition labels as they become older. In addition, respondents with an average monthly salary of NTD 30,000 or more and those who were exercising regularly and were engaged in muscle building, fat loss, or weight control have higher WTP, indicating that those who have higher income, regular exercise, and health goals value products with nutrition labels and are willing to pay more for them. This is consistent with Bleich and Wolfson’s [66] findings, which indicate that consumers with health goals will choose products based on nutrition facts.

The WTP for traceability certification (TAP) manifested significant differences in gender, age, and education level, and the WTP was higher among the respondents who were female, between 30–39 years old, and had college or university education or above. According to the survey results, women, young and middle-aged people, and those who had received higher education attached more importance to the source of ingredients and were willing to pay more for healthy boxed meals with traceability certification. This is in line with Zhang et al. [67], who specified that women are more likely to purchase traceable products than men; in addition, young consumers are more willing to pay more for products with traceable ingredients.

## 5. Discussion

According to the estimation results of the random parameter logit (RPL) models, the respondents were willing to pay extra for healthy boxed meals with traceability certification at a markup of NTD 4.60. This is consistent with Liu et al.’s [68] findings, which indicate that consumers are willing to pay an extra premium of CNY¥1.42 (approximately NTD 6.29) for apples with traceability information, and are willing to pay higher amounts for traceable and safe foods [26,29]. Moreover, in terms of NF, the respondents were willing to pay an additional amount of NTD 4.4. Christoph et al. [7] indicated that people aiming to lose, gain, or control weight for health purposes focus on nutrition facts. Wojcicki and Heyman [19] found that consumers’ emphasis on nutrition facts is related to their healthy eating habits; therefore, nutrition facts can help consumers choose foods that meet their health goals, and consumers prefer healthy boxed meals with nutrition labels, which is consistent with the findings of this study.

In addition, consumers have a significant preference for very good taste and the use of a carbon footprint label. Respondents were most willing to pay extra for very good taste (NTD 4.3), followed by carbon footprint label (NTD 3.3). This is consistent with the literature that when choosing healthy foods, consumers will prefer those with good taste [69,70]. Kähkönen and Tuorila [71] pointed out that if healthy food is tasty and delicious, it can attract consumers and trigger positive emotions, such as pleasure and excitement.

This is consistent with Gadema and Oglethorpe’s [72] findings, which point out that consumers’ knowledge of product labeling affects their decision making. According to Rondoni and Grasso [73], consumers in the UK and Germany have the highest level of concern, understanding, and use of carbon footprint label on food products, while consumers in Poland, Spain, and Sweden have the lowest level of concern. This difference may be related to the timing of the introduction of carbon footprint label in each country.

The results indicate that Taiwanese consumers prefer healthy boxed meals with traceability certification and carbon footprint label. The second target of the SDGs is to “end hunger, achieve food security and improved nutrition, and promote sustainable agriculture.” Currently, Taiwan’s traceability certification system emphasizes the need to maintain environmental sustainability while producing healthy and safe agricultural products. Enterprises in Taiwan have been promoting carbon footprint label since 2009, expecting to strengthen the market competitiveness of low-carbon products with carbon labeling policy and increasing consumers’ awareness of purchasing carbon-labeled products. In addition, in 2022, Taiwan passed the Food and Agricultural Education Act, aiming to deepen the sustainable agricultural programs of food linking agriculture and eat local first. Therefore, it is suggested that the government advocate healthy boxed meal companies to actively obtain traceability certification and carbon footprint label to improve the safety of agricultural products, promote environmental sustainability, and achieve a sustainable consumption and production model in a low-carbon economy.

## 6. Conclusions

### 6.1. Management Implications

The results of this study indicate that consumers are more attracted to and are willing to pay extra for healthy boxed meals that have traceability certification and nutrition facts. Therefore, healthy boxed meal companies should not only focus on the health benefits but also emphasize the traceability certification and nutrition facts of boxed meals to increase the likelihood of consumer purchase. Presently, only a few healthy boxed meal companies in Taiwan use traceability-certified ingredients. Therefore, in order to enhance consumers’ WTP, it is recommended that healthy boxed meal companies introduce traceability certification to achieve consistent production and sales information of ingredients that is open, transparent, and traceable.

Furthermore, considering the growing threat of global warming, the carbon footprint label of products has become one of the tools for governments and enterprises to achieve the target of greenhouse gas reduction. It is suggested that the government should apply the concepts of environmental sustainability and carbon footprint label nationwide to enhance public awareness and encourage healthy boxed meal companies to use carbon footprint label.

The research results indicate that consumers over 40 years old, with an average monthly salary of more than NTD 30,000, who work out regularly, and are in the process of muscle building, fat loss, or weight control have stronger WTP for healthy boxed meals with nutrition labels. This indicates that most consumers who purchase healthy boxed meals are health-conscious individuals. Thus, it is suggested that healthy boxed meal companies cooperate with sports centers and gyms to develop suitable plans according to consumers’ health goals, enhancing the consumption motivation and increasing the turnover of healthy boxed meals.

### 6.2. Research Limitations and Future Research Directions

There are some limitations in the process of this study. The research framework would be more complete if we can expand the research scope in the future. First, this study focuses on five attributes of healthy boxed meals (i.e., taste, nutrition facts, traceability certification, carbon footprint label, and price). In the future, different attributes (e.g., hiring nutritionists to design meals and brand familiarity) can be added to better understand consumers’ preferences for different attributes of healthy boxed meals.

The main limitation of this study is the effects of different dishes and ingredients on the tastes of healthy boxed meals may not be the same, and are difficult to quantify. Moreover, different healthy boxed meal companies may use different magnitudes of ingredients even for the same healthy boxed meals, so such data will increase the systematic error of the statistical analysis.

Second, this study is limited to one type of healthy boxed meal with a small price range. Subsequent researchers should refer to this study to investigate the range of daily spending on eating out, which can be used as a basis for setting product sales prices and conducting product development. Furthermore, more types of boxed meals and products can be included for thematic research to provide more representative reference data for the bento industry.

Next, the participants were consumers who purchased healthy boxed meals in Taiwan; therefore, the extrapolation of the findings is limited. To improve the accuracy of the research results and obtain complete information, subsequent researchers can use this study as a basis and collect data from different countries and regions to understand the determinants of purchased healthy boxed meals across societies and cultures. Finally, the latent class model (LCM) may be used in future research to inspect whether the respondents have heterogeneous preferences for the attribute levels designed in the study to conduct a more comprehensive analysis and discussion of the healthy boxed meals issue.

## Figures and Tables

**Table 1:** Attributes and attribute levels of healthy boxed meals.

Attribute	Levels	Variable Name	Variable Value	Expected Sign	Literature
Taste	(1) Ordinary (2) Good (3) Very good	GD	“−1” means “ordinary” “1” means “good” “0” means “very good”	+	Livingstone et al. [12]
		VGD	“−1” means “ordinary” “1” means “very good” “0” means “good”	+	
Nutrition facts	(1) Not provided (2) Provided	NF	“1” means “provided” “−1” means “not certified”	+	Gracia et al. [54]
Traceability certification	(1) Not certified (2) Certified	TAP	“1” means “certified” “−1” means “not provided”	+	Kumvenji et al. [28]
Carbon footprint label	(1) Not provided (2) Provided	CF	“1” means “provided” “−1” means “not provided”	+	Wong et al. [37]
Price	(1) NTD 99 (2) NTD 99 (NTD 1–5 extra) (3) NTD 99 (NTD 6–10 extra)	FUND	“99” means “NTD 99” “99 + 1” means” NTD 99 (NTD 1–5 extra)” “99 + 6” means “NTD 99 (NTD 6–10 extra)”	–	The Protein Box and Health It chain healthy boxed meal companies

Note: NTD, new Taiwan dollar (1 NTD = 0.033 USD); TAP, traceable agricultural products; CF, carbon footprint.

**Table 2:** Demographic information.

Variable	Description	Sample Size	Percentage	Variable	Description	Sample Size	Percentage
Gender	Male	223	45.1%	Eating out per week	Less than 2	65	13.2%
	Female	272	54.9%		3–5	147	29.7%
Age	20–29	196	39.6%		6–8	117	23.6%
	30–39	177	35.8%		Above 9	166	33.5%
	40–49	61	12.3%	Average spending on eating out per day (NTD)	Less than NT$200	223	45.1%
	50–59	42	8.5%		NT$201–300	189	38.2%
	60 or above	19	3.8%		NT$301–400	52	10.5%
Education level	Junior high school or below	40	8.1%		Above NT$400	31	6.2%
	University and college	351	70.9%	Purchased healthy per week	0	203	41%
	Master	96	19.4%		1–2	179	36.2%
	PhD	8	1.6%		3–4	85	17.1%
Average personal monthly income (NTD)	Less than NT$10,000	70	14.1%		Above 5	28	5.7%
	NT$10,001–30,000	101	20.4%	BMI (kg/m^2^)	<18.5	28	5.7%
	NT$30,001–50,000	225	45.5%		18.5 ≤ BMI < 24	268	54.1%
	NT$50,001–70,000	77	15.6%		24 ≤ BMI < 27	122	24.6%
	NT$70,001–90,000	10	2.0%		27≤	57	11.4%
	Above NT$90,000	12	2.4%		Unknown	20	4.0%

**Table 3:** Estimation results of the RPL models.

Attribute and Variable	RPL				
	Coefficient	*t*-Value	Standard Error	*t*-Value	WTP (NTD)
GD	0.489	−1.35 *	0.404	1.35	3
VGD	0.713	8.16 ***	0.448	3.84 ***	4.3
NF	0.723	6.0 **	0.429	5.13 ***	4.4
TAP	0.747	11.1 ***	0.415	7.97	4.6
CF	0.551	−5.44 **	0.321	5.62	3.3
FUND	0.163	−7.6 *			
Number of choice sets	990				
Log-likelihood ratio	−589.744				

***, **, and * are significant at 1%, 5%, and 10%, respectively; NTD: new Taiwan dollar (1 NTD = 0.033 USD); RPL: random parameter logit; WTP: willingness to pay.

**Table 4:** Comparison of respondents’ socioeconomic background and WTP for the attributes of healthy boxed meals.

Socioeconomic Background		Sample Size	ASC		NF		TAP	
			Average	*t*-Value	Average	*t*-Value	Average	*t*-Value
Gender	Male	223	−22567	−2.37 *	2.7	1.82	3.9	1.39 ***
	Female	272	−26242		3.4		5.6	
Age	20–29 years old	196	−26266	−3.61 *	2.6	1.67 **	2.9	2.49 **
	30–39 years old	177	−26871		2.8		5.3	
	40–49 years old	61	−24081		4.6		4.4	
	50–59 years old	42	−25144		3.0		3.7	
	Over 60 years old	19	−24524		3.1		3.0	
Education level	Senior high/vocational school (inclusive) or below	40	−22534	1.28	2.4	0.86	3.2	2.14 ***
	College or university	351	−26329		3.2		4.4	
	Master’s	96	−26166		3.4		5.1	
	Doctorate	8	−26562		3.0		4.5	
Average personal monthly income	Less than NTD 10,000 (inclusive)	70	−20349	−4.72 **	2.5	2.45 ***	2.7	2.47
	NTD 10,000–30,000 (inclusive)	101	−18663		2.0		3.2	
	NTD 30,000–50,000 (inclusive)	225	−26477		3.2		3.5	
	NTD 50,000–70,000 (inclusive)	77	−26119		4.2		4.1	
	NTD 70,000–90,000 (inclusive)	10	−26563		4.1		4.5	
	Above NTD 90,000	12	−26870		4.1		4.7	
Muscle building, fat loss, or weight control	Yes	319	−26165	0.75	4.7	1.02 ***	3.4	1.88
	No	176	−26059		3.1		3.2	
Regular exercise	Yes	280	−26164	0.75	4.5	1.02 **	3.7	1.88
	No	215	−23081		2.7		3.3	

***, **, and * are significant at 1%, 5%, and 10%, respectively.

## Data Availability

The data that support the findings of this study are available from the corresponding author, H.-S.C., upon reasonable request.
